# Supplementary material for: ToxCast chemical library Wnt screen identifies diethanolamine as an activator of neural progenitor proliferation
Source: FASEB Bioadv. 2022 Mar 28;4(7):441–53. doi: 10.1096/fba.2021-00163 (PMC9254222; doi:10.1096/fba.2021-00163)
Supplement: Supplementary file 1 — Figure S1‐S3 [file FBA2-4-441-s004.docx]

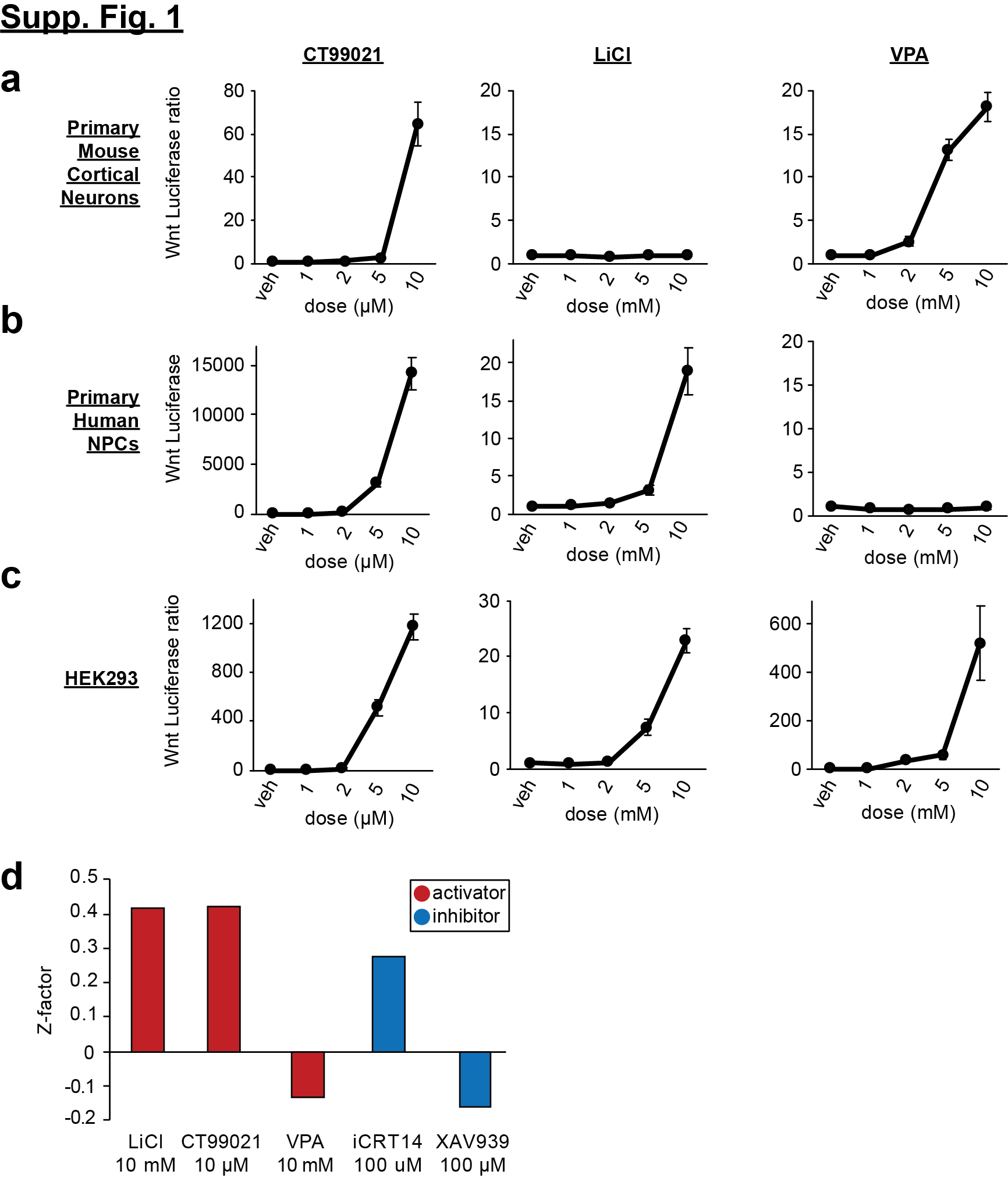


**Supporting Figure 1 – Establishing ToxCast screen conditions**

**a-c)** Chemicals known to activate Wnt signaling tested in primary mouse cortical neurons (**a**), primary human neural progenitor cells (**b**), and HEK293T cells (**c**), without *UBE3A^T485A^* overexpression. Primary cells were transduced with lentiviruses carrying BAR:Firefly and Tk:*Renilla*. HEK293T cells were transiently transfected with plasmids. n=4 per condition.

**d)** Z-factor analysis for known Wnt activating and inhibiting control chemicals at indicated concentrations in HEK293T cells.

**
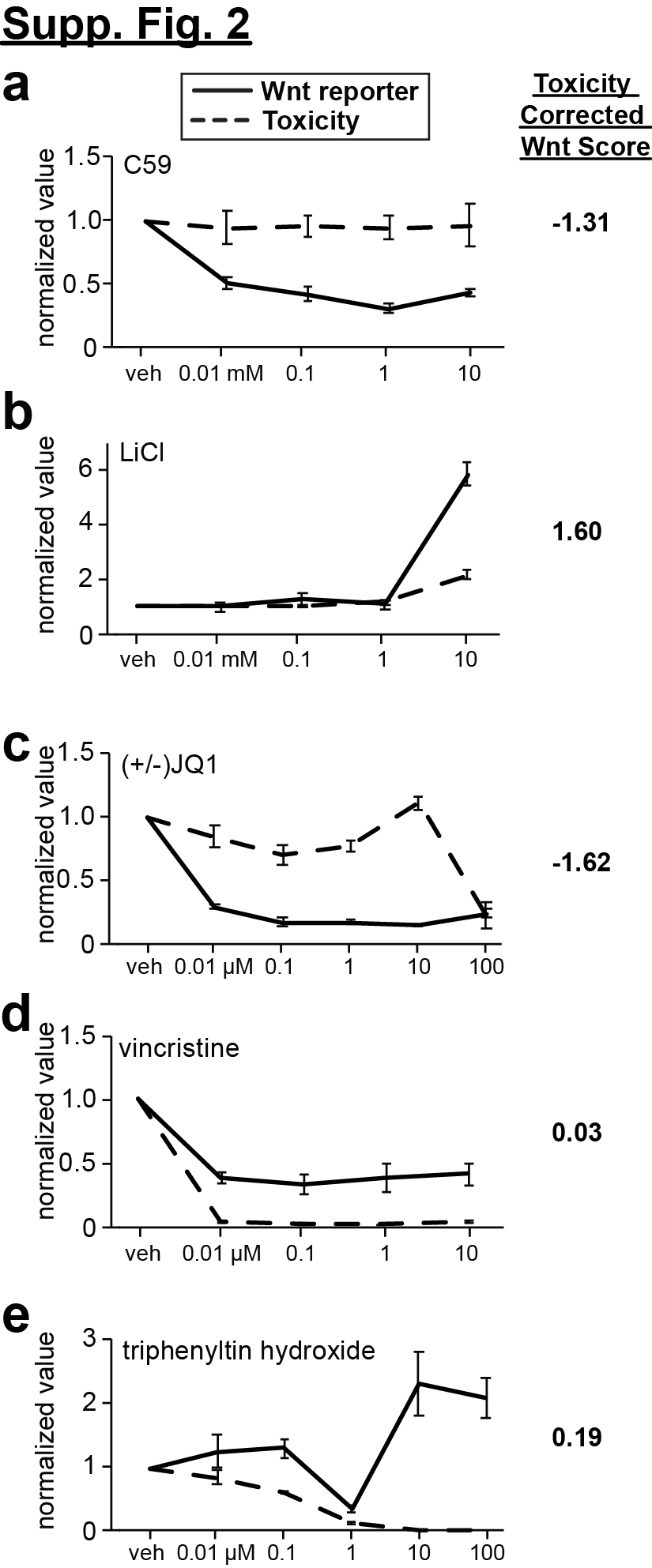
Supporting Figure 2 – Examples of concentration-response curves and Wnt Score in HEK293T cells**

**a-c)** Control chemicals that modulate Wnt reporter (solid line) at concentrations that are non-toxic (dashed line). The Wnt score listed to the right of each graph is a single score combining multiple concentrations of the Wnt reporter values with a penalty for toxicity (see methods).

**d,e)** Concentration-response curves for two ToxCast chemicals that significantly inhibit (**d**) or activate (**e**) the Wnt reporter, but do so at concentrations that are toxic. The Wnt score listed to the right, centered around 0, reflects the penalty incurred on Wnt activation from toxicity.

**
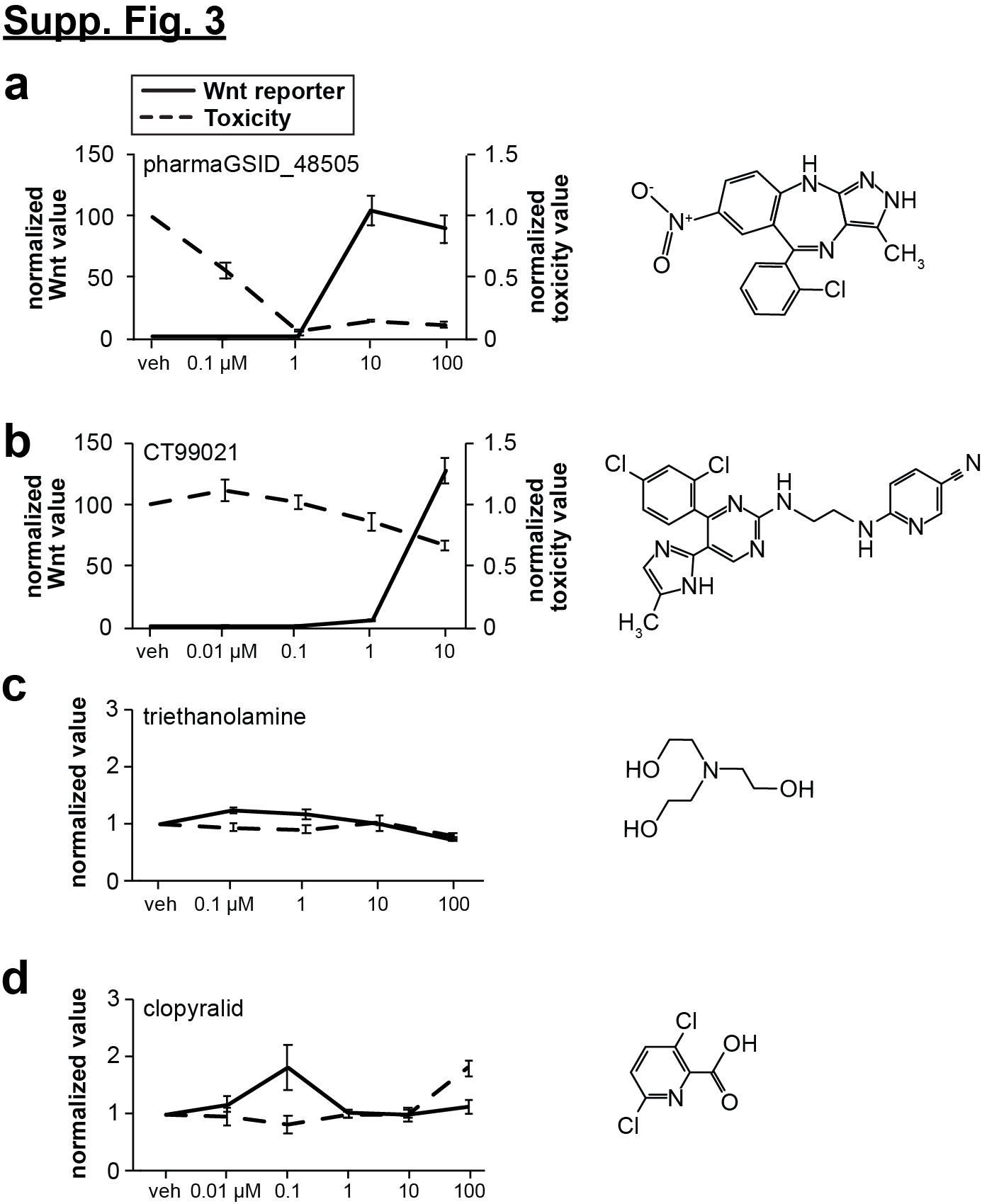
**

**Supporting Figure 3 – Concentration-response curves for two Wnt activating chemicals and two chemicals that are structurally similar to activators, but that fail to activate Wnt signaling.**

**a-d)** HEK293T cells transfected with *UBE3A^T485A^* treated with **(a)** the most potent ToxCast wnt activator pharmGSID_48505, and **(b)** CT99021, the most potent Wnt activator identified to date. Results demonstrate similar effect sizes with higher toxicity for pharmaGSID_48505. **(c)** Triethanolamine, the trimeric form of monoethanolamine, fails to activate Wnt reporter. **(d)** Clopyralid alone also fails to activate Wnt reporter.
